# Case Commentary: Successful use of olorofim for the treatment of multi-drug-resistant *Lomentospora prolificans* infection in a child

**DOI:** 10.1128/aac.01072-25

**Published:** 2025-09-26

**Authors:** Nathan P. Wiederhold

**Affiliations:** 1 Fungus Testing Laboratory, Department of Pathology and Laboratory Medicine, University of Texas Science Center at San Antonio 12346, San Antonio, Texas, USA; Houston Methodist Hospital and Weill Cornell Medical College, Houston, Texas, USA

**Keywords:** *Lomentospora prolficans*, olorofim, multidrug resistance, case report, child

## Abstract

*Lomentospora prolificans* is a multi-drug-resistant filamentous fungus that can cause invasive disease in both immunocompromised and immunocompetent hosts. Current treatment options are limited. Olorofim is an investigational antifungal that demonstrates activity against several different molds, including many that are resistant to other antifungals. The case presented by Fortini et al. (Antimicrob Agents Chemother 69:e00602-25, 2025, https://doi.org/10.1128/aac.00602-25) describes the successful use of olorofim treatment in an 8-year-old who suffered from *Lomentospora prolificans* tendonitis, synovitis, and osteomyelitis.

## COMMENTARY

Fortini et al. (1) describe a case of invasive lomentosporiosis successfully treated with the investigational antifungal olorofim. In this case, an 8-year-old immunocompetent female suffered from *Lomentospora prolificans* tendonitis, synovitis, and osteomyelitis following a traumatic injury to her left knee. Approximately 1 month following the injury, she was started on liposomal amphotericin B but was then transitioned to combination therapy with voriconazole, micafungin, and terbinafine after species identification. Antifungal susceptibility testing demonstrated little to no *in vitro* activity for each of the clinically available antifungals tested, as evident by high MIC/MEC values. In contrast, olorofim had potent activity (MIC 0.06 μg/mL). Due to her age, she did not qualify for enrollment in a clinical trial but was able to receive olorofim as compassionate use. Improvement was noted within 16 days of the start of olorofim, and she continued olorofim for a total of 6 months with no recurrence 2 years after her initial diagnosis. This is the first report of successful use of olorofim in a child.

*Lomentospora prolificans* was first described in 1974 (2), and the first reported human infection was reported 10 years later, cultured from a bone biopsy specimen collected from a patient with osteomyelitis (3). This species has been known by different names over the years and was most recently known as *Scedosporium prolificans* before being transferred back to the genus *Lomentospora* and again called *L. prolificans* in 2014 (4, 5). It is capable of causing invasive disease in both immunocompetent and immunocompromised individuals, thus acting as both a primary and opportunistic pathogen (6–8). Because of its pan-resistance to clinically available antifungals and its ability to cause disease in both immunocompromised and immunocompetent patients, the World Health Organization placed *L. prolificans* on its 2022 Fungal Priority Pathogens list (9). This pathogen is capable of causing a wide variety of infections. Depending upon the host status and the route of entry into the body, infections can be localized, extend to surrounding tissues, or disseminate to other organs (6, 8, 10). Mortality has been reported as high as 87.5% in disseminated disease (6). Skin, soft tissue, muscle, bones, and joints are common sites of infection in immunocompetent hosts.

Because of its intrinsic resistance to clinically available antifungals, treatment of *L. prolificans* infections is challenging. Clinical guidelines recommend combination therapy including voriconazole and terbinafine, and that surgical resection occur when feasible (11). The combination of voriconazole plus terbinafine is based on the results of a retrospective review of 41 patients from the FungiScope registry (12). In this study, voriconazole plus terbinafine was associated with a higher rate of treatment success (63% with this combination versus 9% with other regimens). A higher probability of survival was also reported with this combination compared to other treatments (median survival 150 days vs. 17 days, respectively).

Olorofim (formerly F901318, F2G, Ltd) is a first-in-class member of a novel class of antifungals, the orotomides. It acts as a reversible inhibitor of dihydroorotate dehydrogenase, which is involved in the *de novo* synthesis of pyrimidine (13). Inhibition of pyrimidine biosynthesis suppresses uridine-5′-monophosphate (UMP) and uridine-5′-triphosphate formation, both of which are important for several cellular processes (14–16). Numerous studies have demonstrated that olorofim has potent *in vitro* activity against different molds, including those that often have reduced susceptibility or resistance to other antifungals, including azole-resistant *A. fumigatus, Scedosporium* spp., *L. prolificans, Microascus/Scopulariopsis,* and members of the *Rasamsonia argillacea* species complex, among others (13, 17–31). Potent activity has also been reported against dimorphic fungi (13, 21, 32, 33), while the activity of olorofim against *Fusarium* is species-dependent (13, 27, 28, 34, 35). This *in vitro* activity has also translated into *in vivo* efficacy in different animal models of invasive mycoses, including those caused by different *Scedosporium* spp. and *L. prolificans* (13, 20, 33, 36, 37). However, olorofim lacks activity against yeasts, members of the order Mucorales (the causative agents of mucormycosis), and some dematiaceous fungi, such as *Alternaria alternata* (13, 29).

Previous case reports have shown successful outcomes in patients with invasive lomentosporiosis treated with olorofim. One involved a 56-year-old female who developed disseminated infection status post-chemotherapy for T-cell acute lymphoblastic leukemia, which included fungemia, endophthalmitis, and involvement of the lumbar spine, lungs, and aortic valve (38). Therapy with voriconazole plus terbinafine as well as surgical debulking of the spine had failed. Olorofim monotherapy was begun 11 months into the infection, and within 6 months improvement was observed without adverse effects. In a second case, a 49-year-old female developed lomentosporiosis of the right breast implant, which spread to the soft tissues, ribs, and sternum. Treatment failed despite implant removal, repeat debridement, and multiple antifungals, including voriconazole, terbinafine, miltefosine, posaconazole, and anidulafungin (39). She began olorofim, which continued for 322 days with gradual healing of the surgical site and subsequent wound closure.

Recently, results were published from a multicenter, single-arm phase 2b study that evaluated the efficacy of olorofim in patients 16 years of age or older with few or no treatment options for proven or probable invasive infections (40). This included 26 patients with invasive disease caused by *L. prolificans*. The primary endpoint, global response at day 42, was achieved in only 28.7% of patients, with a response rate of 42.3% in those infected with *L. prolificans*. However, when stable disease was classified as success, global responses were 72.5% at day 42 and 63.4% at day 84. Against lomentosporiosis, response rates were 76.9% and 73.1% at days 42 and 84, respectively, with all-cause mortality at 11.5%. In the retrospective study that reported improved outcomes with the combination of voriconazole and terbinafine, stable disease was also considered a success (12).

As the current cases, previous case reports, and phase 2b study demonstrate, olorofim is a welcome option for the treatment of *L. prolificans* infections. However, it is not a panacea for difficult-to-treat mold infections. In the phase 2b study, 20 (10%) patients had hepatic adverse events that were at least possibly related to olorofim, of which five were considered serious (40). This is important given the duration of treatment that may be necessary for such cases. Olorofim is metabolized by several CYP 450 enzymes and is also a known inhibitor of CYP3A4; thus, drug interactions are possible as with the azoles (41, 42). It is also unclear at this point if therapeutic drug monitoring will be required for olorofim. In this case report, two olorofim trough concentrations were reported, each being above the threshold (>0.1 µg/mL) currently considered necessary for efficacy (37). Given its unique spectrum of activity, species or genus-level identification will be needed to determine if olorofim is an appropriate option.
